# Seroepidemiological Survey of West Nile Virus Infections in Horses from Berlin/Brandenburg and North Rhine-Westphalia, Germany

**DOI:** 10.3390/v14020243

**Published:** 2022-01-25

**Authors:** Felicitas Bergmann, Dagmar S. Trachsel, Sabita D. Stoeckle, Joke Bernis Sierra, Stephan Lübke, Martin H. Groschup, Heidrun Gehlen, Ute Ziegler

**Affiliations:** 1 Friedrich-Loeffler Institut, Federal Research Institute for Animal Health, Institute of Novel and Emerging Infectious Diseases, 17493 Greifswald-Insel Riems, Germany; felicitas.bergmann@fli.de (F.B.); martin.groschup@fli.de (M.H.G.); 2 Department of Veterinary Medicine, Equine Clinic, Internal Medicine, Freie Universität Berlin, 14163 Berlin, Germany; dagmar.trachsel@vetmeduni.ac.at (D.S.T.); sabita.d.stoeckle@fu-berlin.de (S.D.S.); Heidrun.Gehlen@fu-berlin.de (H.G.); 3 Equine Veterinary Practice, 53819 Neunkirchen-Seelscheid, Germany; Joke.BernisSierra@cvua-rrw.de (J.B.S.); dr.stephan.luebke@tierarztpraxis-luebke.de (S.L.)

**Keywords:** West Nile virus, horses, seroepidemiological, seroprevalence, Germany

## Abstract

Following the introduction of the West Nile virus (WNV) into eastern Germany in 2018, increasing infections have been diagnosed in birds, equines, and humans over time, while the spread of WNV into western Germany remained unclear. We screened 437 equine sera from 2018 to 2020, excluding vaccinated horses, collected from convenience sampled patients in the eastern and western parts of Germany, for WNV-specific antibodies (ELISAs followed by virus/specific neutralization tests) and genomes (RT-qPCRs). Clinical presentations, final diagnoses, and demographic data were also recorded. In the eastern part, a total of eight horses were found WNV seropositive in 2019 (seroprevalence of 8.16%) and 27 in 2020 (13.77%). There were also two clinically unsuspected horses with WNV-specific antibodies in the western part from 2020 (2.63%), albeit travel history-related infections could not be excluded. None of the horse sera contained WNV-specific genomes. Eight horses in eastern Germany carried WNV-IgM antibodies, but only four of these showed typical clinical signs. These results underline the difficulty of detecting a WNV infection in a horse solely based on clinical signs. Thus, WNV circulation is established in the horse population in eastern Germany, but not yet in the western part.

## 1. Introduction

West Nile virus (WNV) is a mosquito-borne arbovirus, which is present on every continent in the world, except Antarctica [1]. WNV is a member of the family *Flaviviridae* [2] and a threat to animal and human health [3]. The virus circulates in an enzootic cycle between ornithophilic mosquitoes as vectors and various avian species [4,5]. It has been found in over 150 bird species [6], passerines serve as the primary reservoir hosts, and corvids and birds of prey are often affected by severe clinical disease [7,8]. A variety of mammalian and reptilian species are also susceptible to WNV infection under natural conditions.

The majority of susceptible vertebrates (excl. fish) do not develop viremia levels high enough to support virus transmission and therefore, are considered dead-end hosts [9,10]. Infections themselves are often asymptomatic. Horses and humans can seroconvert without clinical signs/symptoms. However, both species can also develop clinical diseases, ranging from mild febrile illness (West Nile fever) to encephalitis with a fatal outcome [11,12]. Neurological signs were recorded in about 8% of naïve infected horses in California [13]. The type and severity of neurological signs largely depend on the extent of the central nervous system involvement and the location of the damage [14,15]. Neuroinvasive disease is characterized by ataxia, encephalitis, muscle fasciculation of the head (including the muzzle) and neck, lethargy, and weakness combined with hyperesthesia and in some cases, also cranial nerve deficits, such as dropping lips [14,16,17,18,19,20,21,22,23]. Aside from fever, which is not a typical sign but rather an inconsistent finding in WNV-infected horses, depression, anorexia, colic, or abnormal gait often are the first recognized signs [24]. While clinically affected horses may recover, neurological signs often persist lifelong [13,17]. The fatality rate in horses suffering from neurological signs is high and ranges from 22% to 44% [16,20,25]. Vaccinated horses show less severe clinical signs and a better prognosis for a full recovery without persisting neurological signs [13,17,24,26].

To date, there is no specific anti-viral treatment, and apart from vector control strategies around horse stables, vaccination is the best prophylaxis. Three different vaccines are licensed in the European Union and are available in Germany [27]. These products can protect horses against the development of a clinical manifestation and effectively prevent long-lasting neurological impairments [24,26,28,29,30].

Since the introduction of WNV lineage 2 from Africa into Europe, i.e., into Hungary in 2004 [31], this virus lineage spread to neighboring countries, particularly the Mediterranean and Balkan countries, e.g., Austria, Greece, Italy, Serbia, and Romania (summarized by [32,33]), within a few years. This was accompanied by an increase in the severity and incidence of clinical cases in humans and equines [34,35,36]. Since 2017, WNV lineage 2 has spread farther westwards causing massive outbreaks in southern/south-eastern and even central European countries in 2018. The number of confirmed human cases in 2018 was 7.2-fold higher when compared to the previous year. Two hundred and fifty-eight horses were affected, and WNV was found in more countries than in the preceding transmission seasons [37]. Optimal environmental conditions, especially unusually high temperatures in 2018, presumably led to a decreased extrinsic incubation period for WNV in vector competent mosquitoes. High temperatures in combination with water shortage could have forced the mosquitoes to seek out human habitats increasing the frequency of mosquito–human contact. This, in turn, led to rapid virus amplification and a greater transmission risk for vertebrates in Europe [3].

In 2018, WNV lineage 2 was first detected in resident, wild, and aviary birds and two horses in Germany [38]. In 2019, an epizootic emergence of WNV was observed again and the number of infected birds and horses was once again considerably higher (76 birds and 36 horses) compared to 2018 (12 birds and 2 horses). WNV hotspots were located in the eastern part of Germany (federal states: Saxony, Saxony-Anhalt, Berlin, and certain regions in Brandenburg) [39,40]. Furthermore, in autumn 2019, the virus was found for the first time in mosquitoes of the *Culex pipiens* complex, which is known as a potential vector for WNV in Germany [41]. Five human autochthonous cases were also confirmed for the first time in WNV endemic regions in Germany. The WNV epizootic continued in 2020, and a multitude of birds and horses were affected in the already known impacted areas of Eastern Germany [42]. In addition, a significant increase in human WNV cases was recorded in 2020, including the first fatal case [43,44]. Currently, WNV is spreading farther northwards, with a recent publication on the first detection of WNV in the Netherlands in mosquitoes, birds, and humans [45,46].

Data concerning WNV monitoring of horses in Germany are limited, only two studies have been conducted to date [47,48]. Both publications could not verify an endemic circulation of WNV in Germany as no WNV-specific antibodies were detected in the tested horses. Only individual serological cross-reactions with specific tick-borne encephalitis virus antibodies were described.

While WNV reached Germany in 2018, with notifiable cases in birds and horses, no recent information on the serological situation in horses is available. Therefore, this study intended to elucidate the actual WNV situation by screening horses in two different regions in Germany, one in the eastern part (Berlin/Brandenburg, panel A), the current WNV endemic region, and another one in the western part (North Rhine–Westphalia, panel B), to date without detected WNV circulation. Another question was how well acute WNV infections in horses can be detected and WNV-associated clinical signs can be determined.

## 2. Materials and Methods

### 2.1. Study Design and Sample Collection

A total of 437 serum samples from horses were arbitrarily collected from two regions in Germany in three consecutive years (2018-2020). In detail, in the Berlin/Brandenburg (BE/BB) region, panel A, we collected a total of 98 serum samples in 2019 and 296 in 2020. In North Rhine–Westphalia (NRW), panel B, we collected 67 in 2018 and 76 serum samples in 2020. A sampling of panel A was performed by the equine clinic of the department of veterinary medicine in Berlin and that of panel B by an equine ambulatory practice from Neunkirchen-Seelscheid (NRW). Blood samples were taken as part of clinical work-ups or during health checks of patients of the participating institutions. The only inclusion criterion was that the horses had to be kept in Germany. With the exception of NRW in 2018, where the samples were collected year-round, sampling started in June (BE/BB)/July (NRW), at the beginning of the mosquito and WNV season, and lasted until the end of the year.

The samples were taken consecutively, independent of the reason for admission. Care was taken to ensure even distribution throughout the sampling periods. However, there was no formal randomization and sampling remained at the discretion of the clinician in charge. In order to compare samples between the two institutions, the specific breed of the horses was recorded and categorized as follows: Warmblood or Warmblood-crossbred (WB), Draft Horse or Draft Horse-crossbred (DH), American Quarter Horse, American Quarter Horse-crossbred or related breed (AQH-type), Thoroughbred (THB), Standardbred (STB), Arabian, Arabian-crossbred or related breed (Arabian-type), or Pony. Furthermore, the age and sex (castrated male, C; intact male, M; or female, F) of the horses were recorded.

### 2.2. Classification of Clinical Signs and Diagnoses

For a detailed description of the study samples, the reason for presentation was noted as well as the final diagnosis of internal medicine-related diseases. The reason for presentation was classified as follows: presented with clinical signs typically related to internal medicine (group IM, for example, cough, nasal discharge, abdominal pain or other gastrointestinal related issues, endocrinological dysregulation, neurological signs, ophthalmological diseases, dermatological or urogenital diseases, unspecific signs demanding an internal medical work-up, such as fever of unknown origin, etc.), presented with musculoskeletal problems (group MS, for example, lameness evaluation, elective surgery), presented for a dental investigation (group dental), or finally, the sample was taken as part of a routine health check, and the horse was otherwise completely healthy (group healthy).

In the IM group, medical records were reviewed, and final diagnoses were documented by the treating clinicians during consultation. Clinical signs in horses for which an acute WNV infection was detected by IgM ELISA are reported separately.

### 2.3. Molecular Investigations

RNA isolation from serum was performed using the Viral RNA Mini Kit (Qiagen, Hilden, Germany) following the manufacturer’s instructions. The quantitative reverse transcription-polymerase chain reaction (RT-qPCR) for WNV was carried out according to a previously published protocol using primers and probes which target 118 base pairs in the 5′-untranslated region (UTR) [49]. Furthermore, a RT-qPCR for Usutu virus (USUV) and a RT-qPCR for tick-borne encephalitis virus (TBEV) were performed to exclude all relevant zoonotic flaviviruses in these areas [50,51]. Additionally, an internal control system, IC2, as a duplex real-time PCR, was included [52].

### 2.4. Serological Investigations

All sera were screened for WNV-specific antibodies using a commercially available competition ELISA, which allowed species-independent recognition of WNV antibodies against the Pr-E envelope protein, following the manufacturer’s instructions (ID Screen^®^ West Nile Competition, IDVet, Montpellier, France). Additionally, to detect recent WNV infections in horses, a commercially available IgM capture ELISA from the same commercial provider was used.

As the WNV competition ELISA detects flavivirus antibodies, sera with a reactive result (positive or doubtful) were additionally verified by means of virus neutralization tests (VNT). VNTs are the gold standards in identifying WNV or cross-reactivity in serological diagnostics.

The VNT for WNV was performed under biosafety level 3 conditions and using Vero cells on 96-well plates as described earlier [53,54]. Some minor modifications were implemented for the relevant virus strains. We used WNV strain Austria (lineage 2, GenBank accession no. HM015884, kindly provided by S. Revilla-Fernandez, AGES Mödlingen, Austria) for the horse samples from 2018 and 2019, and WNV strain Germany (lineage 2, GenBank accession no. MH924836) for samples from 2020. To exclude cross-reacting antibodies among the Japanese encephalitis serogroup, we used USUV strain Germany (Europa 3, GenBank accession no. HE599647) and for the tick-borne encephalitis serogroup, the TBEV strain Neudoerfl (kindly provided by G. Dobler, Bundeswehr Institute of Microbiology, Munich, Germany, GenBank accession no. U27495), following the identical protocols. The neutralizing antibody titer, i.e., the neutralization dose 50% (ND_50_) of a sample was defined as the maximum dilution at which the cytopathic effect was inhibited in 50% of the wells and was calculated according to the Behrens–Kaerber method [55]. Serum samples with ND_50_ values above 10 were considered positive or seropositive, and samples with ND_50_ values lower than 10 as negative or seronegative. Sera were considered specific when only one of the viruses was neutralized or the ND_50_ titers were ≥ 4 times higher for one of the viruses.

### 2.5. Statistical Analyses

The distribution of breeds, sex, reasons for presentation, and final diagnoses in the two study samples was compared with a Fisher’s exact test.

The distribution of ages in the study samples at both institutions did not correspond to a normal distribution (Shapiro–Wilk normality test). Therefore, the ages of the included horses were compared with a Mann–Whitney test between the institutions and between the years of sampling at each institution. Further, a Kruskal–Wallis test with a Dunn’s Multiple Comparison Test was used to compare different groups of seropositivity. 

Univariate logistic regression models were used to assess the association between age, sex, breed, year, and site of sampling and the outcome (being seropositive). We used a stepwise forward selection to construct multivariable models. The selection of relevant variables was based on the likelihood ratio test.

For the statistical analyses, a commercially available software was used (Microsoft Excel 2013, Microsoft Corporation, Redmond, WA, USA, GraphPad Prism^®^, version 5.01, GraphPad Software, San Diego, CA, USA, R-software^®^, version 3.1.0, R Development Core Team, Vienna, Austria). A *p*-value of less than 0.05 was deemed statistically significant. Seroprevalence and 95% confidence intervals (95% CI) were calculated with R version 4.0.3.

### 2.6. Maps

A GIS-Analysis of the geographical location of the horse samples in combination with the serological results was performed by using the ArcGIS ArcMap 10.8.1 software (ESRI, Redlands, CA, USA) and © GeoBasis-DE/BKG 2020.

### 2.7. Ethical Statement

Blood samples were collected during obligatory routine diagnostic schemes for other clinical aspects or diseases by the equine clinic or equine practice, and the remaining serum material was provided for our study. Therefore, the sampling of horses included in this study was not classified as an animal experiment and no further approvals were necessary.

## 3. Results

### 3.1. Analyses of the Specific Breed, Sex, and Age of the Horses Studied

The demographic distribution of the horses concerning breed and sex is depicted in the Supplementary Materials Data: Figures S1a and S2a. Fisher’s exact test revealed no significant differences in the breed and sex distribution of the sampled horses between BE/BB and NRW. Supplementary Materials Data: Figures S1b,c and S2b,c give a detailed overview of the demographic features (breed and sex) per year and panel.

**Figure 1:**
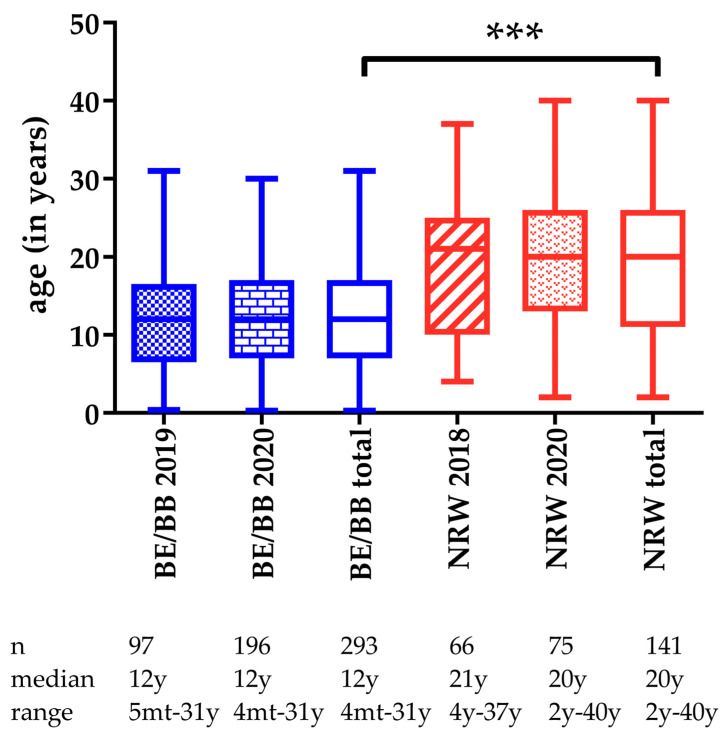
Box plots (median and range) showing the age distribution in the sampled horses from the regions Berlin/Brandenburg (BE/BB) and North Rhine–Westphalia (NRW) (*n* total = 434, *n* in NRW 2018 = 66, *n* in NRW 2020 = 75, *n* in BE/BB in 2019 = 97, *n* in BE/BB in 2020 = 196, for 3 horses the age had not been reported). ***, *p* ≤ 0.0001; Berlin/Brandenburg (BE/BB) and North Rhine–Westphalia (NRW), mt, months, y, years.

The distribution of ages is shown in Figure 1. There were no differences in the ages between panel B sampled in NRW 2018 and 2020 (Mann–Whitney test *p* = 0.43) or panel A in BE/BB 2019 and 2020 (*p* = 0.92). However, the horse population in panel B (NRW) with a median age of 20 years was significantly older than the sampled horses in panel A (BE/BB) with a median age of 12 years (*p* ≤ 0.0001).

### 3.2. Interpretation of Clinical Signs and Diagnoses

The reasons for presentation leading to the final diagnosis of internal medicine-related problems are reported in Table 1. The reason for presentation differed significantly when comparing NRW 2018 and 2020 (Fisher’s exact test 0.01), BE/BB 2019 and 2020 (*p* = 0.0005), or NRW and BE/BB (*p* = 0.0005). Furthermore, the final diagnosis of internal medicine-related problems differed between the years and the institution (NRW 2018 to 2020 *p* = 0.01, BE/BB 2019 to 2020 *p* = 0.005, NRW to BE/BB *p* = 0.0005).

### 3.3. Molecular Results

A total of 370 sera (294 samples from panel A, and 76 samples from panel B) were tested by RT-qPCRs for WNV, USUV, or TBEV genomes. No specific RNA from these three viruses was detected (Table 2). WNV did not arrive in eastern Germany until the end of August 2018, the serum samples from NRW 2018 were not tested by molecular methods.

### 3.4. Serological Results

Flaviviruses are structurally highly similar, leading to cross-reacting antibodies upon infection, which compromises the commercial competitive ELISA. Therefore, positive ELISA results need to be reconfirmed by flavivirus-specific assays. Hence, we tested all horse sera with reactive IgG ELISA results (positive or doubtful) with a virus neutralization test (VNT) against WNV, USUV, and TBEV. Only the reactive ELISA sera that were also confirmed by one of the 3 VNTs were included in the scoring as serologically positive.

#### 3.4.1. ELISA

All 437 serum samples were investigated using the competitive WNV ELISA. The ELISA results showed a high rate of reactive horse sera in panel A from the region Berlin/Brandenburg, in total 46 horses. Only 14 sera of the 2019 samples had a positive (12) or a doubtful (2) ELISA signal. In the samples from 2020, an increase to 31 positive sera and one doubtful serum was determined. In contrast, the ELISA results from panel B (NRW) were considerably lower. There were only two positive ELISA results in 2018 and three in 2020 as well as one doubtful serum (Table 2).

Furthermore, all 437 sera were tested by an IgM ELISA to detect recent WNV infections in horses. This capture ELISA detects WNV-specific IgM antibodies in horse sera up to three months post-infection. Interestingly, panel B from NRW contained no IgM positive results. In contrast, in panel A, we identified two IgM-positive horses in 2019 and six IgM-positive horses in 2020 (BE/BB) (Table 2).

Subsequently, all ELISA reactive samples were investigated further by the different virus neutralization tests (VNT) to exactly determine the virus resulting in ELISA seropositivity.

#### 3.4.2. Virus Neutralization Test (VNT)

ELISA results were confirmed by VNT results, mostly with WNV-specific antibodies (Table 3).

In detail for panel A (BE/BB): Of the 14 sera investigated in 2019, we confirmed eight samples by WNV-VNT, one by TBEV-VNT, and one by USUV-VNT. The remaining four reactive ELISA sera (two positive and two doubtful) were negative in all three VNTs and could not be evaluated. In 2020, of the 32 reactive IgG ELISA samples (31 × positive, 1 × doubtful) we confirmed 27 with WNV-specific antibodies and three by TBEV-VNT. For the remaining two samples the VNT, results of all three tests were negative. Furthermore, among the eight IgM-positive horses, we detected seven horses with a positive competitive WNV ELISA result, which was confirmed by VNT with specific WNV neutralizing antibodies. 

In detail for panel B (NRW): In 2018, the two positive ELISA samples could be confirmed as TBEV-specific antibodies. In 2020, out of three positive and one doubtful ELISA samples, we detected two with WNV-specific antibodies and two with TBEV-specific antibodies.

**Figure 2:**
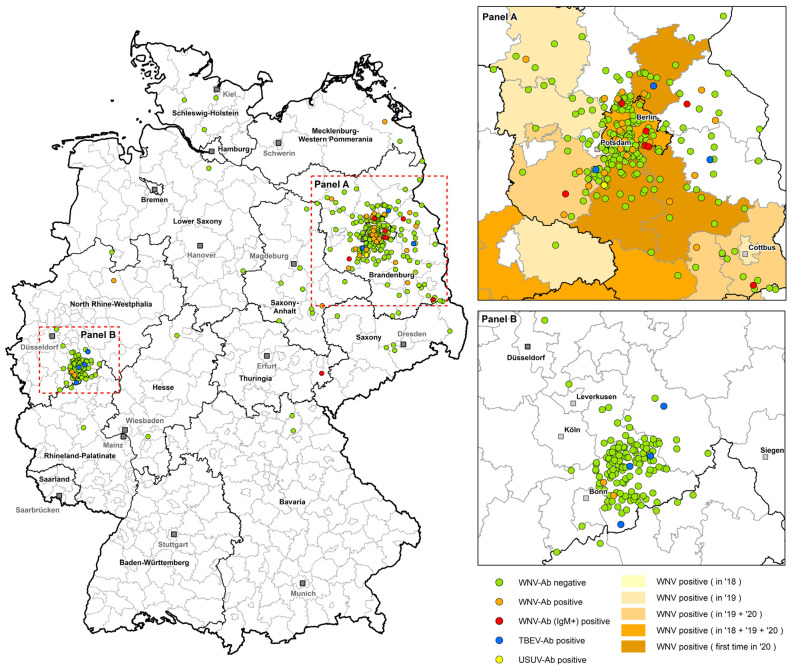
Geographical distribution of the investigated horse sera from each panel (panel A sampled in Berlin/Brandeburg, panel B in North Rhine–Westphalia) and their serological results. Green dots = WNV antibody-negative horses (comp. ELISA negative); orange dots = WNV antibody-positive horses (comp. ELISA and VNT positive); red dots = WNV antibody-positive horses (additionally IgM-positive); blue dots = TBEV antibody-positive horses and yellow dots = USUV antibody-positive horses. In the magnified area from panel A (BE/BB), the endemic WNV areas are highlighted in different shades of orange, which represent the district levels where WNV positive birds and horses were detected in Germany from 2018 to 2020.

Overall, WNV seroprevalence (ELISA positive and determined by WNV-VNT) in panel A (BE/BB) was 8.16% (95% CI: 3.59–15.45) in 2019 and slightly increased to 13.77% (95% CI: 9.27–19.41) in 2020. In comparison, the WNV seroprevalence in panel B (NRW) was 0% in 2018 and increased to 2.63% (95% CI: 0.32–9.18) in 2020 (Table 3). The overview of the serological results in our two panels and the geographical distribution throughout Germany is pictured in Figure 2. The ELISA results for each horse and the relevant VNT titers, as well as the final differentiation of the serological results, are presented in the Supplementary Materials (Tables S1–S4).

With an increasing number of WNV infections in Germany, the number of vaccinated horses has also risen. For this reason, we tested an additional 20 vaccinated horses in 2020. In panel B (NRW), we tested four vaccinated horses, and in panel A (BE/BB), 36 vaccinated horses. Interestingly, in panel B (NRW), only two out of four vaccinated horses were positive by competition ELISA, although they could not be confirmed by means of WNV-VNT. 

In panel A (BE/BB), out of 36 vaccinated horses, four horses were tested negative and one doubtful, while 31 horses were tested positive by WNV competition ELISA. A confirmation by WNV-VNT was possible for 20 horses. A complete list of the ELISA and VNT results as well as the vaccination history of the vaccinated horses can be found in the Supplementary Materials (Tables S5 and S6).

### 3.5. IgM Positive Horses and Their Observed Clinical Signs

In panel B (region NRW), no recent WNV infection could be detected since RT-qPCR and WNV IgM ELISA were negative. In panel A (BE/BB), we found two IgM-positive horses in 2019. One horse (case 1) showed minimal neurological signs (e.g., abnormal behavior, muscle fasciculation, hyperesthesia), anorexia, and colic-like signs. The investigation for a WNV infection was carried out in the framework of differential diagnostics. The other horse (case 2) was only detected retrospectively during our surveillance study because unspecific fever and respiratory signs were not associated with WNV infection. In 2020, we detected six IgM-positive horses, three of them during the clinical hospitalization period. These three horses (cases 3, 5, 6) showed neurological signs to varying degrees, whereas one horse had to be euthanized due to progressive clinical severity of neurological signs (case 5). The investigation for a WNV infection proceeded in the context of differential diagnostics. The remaining three horses (cases 4, 7, 8) again were detected retrospectively by our surveillance study. Clinical signs were not related to a neurological virus infection. One horse had been presented in the emergency service due to acute abdominal pain and was taken to laparotomy. The final diagnosis was an incarceration of the small intestine by a lipoma pendulans (case 4). One horse was presented for extraction of a cheek tooth due to a previously diagnosed alveolitis (case 7), and one horse was referred for lameness evaluation and subsequently, a fracture of a coffin bone was diagnosed and treated (case 8). In these three cases, neither the owner nor the clinicians in charge noticed any signs during hospitalization that might indicate a recent WNV infection.

All IgM-positive horses had no WNV vaccination history, and for seven horses, WNV-specific antibodies were also confirmed by VNT (titers range from 60 ND_50_ to 960 ND_50_). Furthermore, all clinical signs of the eight IgM-positive horses are summarized in Table 4.

### 3.6. Relationship of WNV Seropositivity to Age, Breed, and Sex

**Figure 3:**
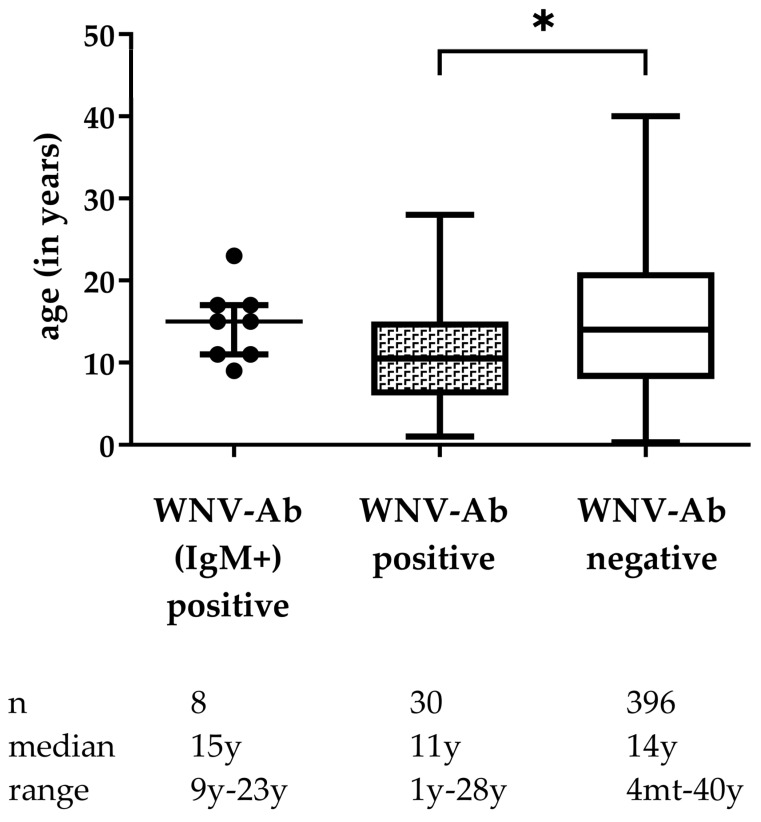
Box plots (median and range) showing the distribution of age in horses that were positive for both IgM and WNV antibodies (Ab (comp. ELISA and VNT positive) *n* = 8, positive for WNV Ab only (comp. ELISA and VNT positive) *n* = 30 and negative for WNV Ab (comp. ELISA negative) *n* = 396 in all horses sampled., for 3 horses the age had not been reported. * *p* < 0.05, mt, months, y, years.

Fisher’s exact test showed that the distribution of breed or sex did not differ significantly between the seropositive horses compared to the seronegative horses (*p =* 0.35 and *p =* 0.58). Seropositive horses showed a lower median age than the seronegative horses. (Figure 3, *p =* 0.04).

The univariate logistic models revealed a significant influence of age, breed, and region of sampling, but none of sex or year of sampling. Older horses were less likely to be seropositive than younger horses. The odds ratio was 0.95 (95% CI 0.91–0.99) per year of age. Ponies were more likely to be seronegative in comparison to WB. The odds ratio to be seropositive was 0.43 (95% CI 0.16–1.03) in comparison to WB. Further, the odds ratio for the site of sampling NRW was 0.10 (95% CI 0.02–0.34).

However, applying a forward selection procedure, the likelihood ratio tests showed that only age and site of sampling were important predictors for seropositivity. 

## 4. Discussion

Since the mid-1990s, the epidemiology and transmission dynamics of WNV have changed in Europe and the virus has been circulating annually in the Mediterranean region [32,33,56]. In 2018, a fulminant increase in the number of human and equine cases was recorded in Europe, exceeding the total number of reported cases in previous seasons. In addition, there was a geographical expansion of WNV, with the first introduction of the virus to Germany [38]. In the preceding years, a continuous monitoring system covering both live and dead wild birds had demonstrated the absence of WNV [54,57,58].

In contrast to wild birds, horses had not been subjected to a systematic WNV monitoring program in the last years, and only two studies with limited results investigated WNV in horses from Germany. One was a risk-based monitoring approach based on the background of the equine infectious anemia situation in the EU in 2010/2011, focusing primarily on the collection of blood samples from deceased horses in rendering plants for animal by-products [48]. The other study from 2007 to 2009 combined sampling of wild birds and free-ranging poultry in specific regions and included only a limited set of samples of horse sera [47]. 

The results from this study give an indication of the WNV seroprevalence in the horse population in the two tested federal states (BE/BB and NRW) keeping in mind, however, that the exact size of the horse population is not known. This is possible due to the large number of samples analyzed from two regions that receive patients spread throughout the respective states/districts (Figure 2).

Therefore, the seroprevalence study presented here focused on the prevalence of WNV and its clinical features in horses. Using 437 horse sera from sampled horses, combined with data on their origins, clinical courses, and final diagnoses, we can show that in the eastern part of the country (panel A), the WNV seroprevalence increased slightly from 8.16% (95% CI: 3.59–15.45) in 2019 to 13.77% (95% CI: 9.27–19.41) 2020 and in panel B, from 0% in 2018 to 2.63% in 2020 (95% CI: 0.32–9.18) (Table 3, Figure 2). However, our logistic regression model (univariate and multivariate) showed that horses in panel B were significantly less likely seropositive than horses in panel A. These results are in accordance with bird surveillance studies [38,39,59], which demonstrated the presence of WNV infection in resident and zoo birds only from the eastern part of Germany. 

Two horses (giving a seroprevalence of 2.63% (95% CI: 0.32–9.18)) from North Rhine–Westphalia (panel B) in 2020 also tested positive for WNV antibodies. For both horses, a travel history cannot be excluded. One horse originally came from Austria and was introduced to the NRW region five years earlier. Since this horse was not used in professional sports events, it probably stayed in NRW. Since the other horse, a race-horse, had stayed in France until 1.5 years earlier, the WNV antibodies could be associated with France. Neutralizing WNV antibodies from a natural infection can persist for at least 15 months [20]. For our clinical case no. 1 (Table 4) from BE/BB, we were able to demonstrate that neutralizing antibodies remained at a high level for more than seven months after natural infection, before it was vaccinated [60]. For panel A, we also do not have detailed information on travel histories outside Berlin/Brandenburg. Therefore, for the two WNV antibody-positive horses, one from Mecklenburg–Western Pomerania (Anklam region) in the northern part of Germany, and one from NRW in the western part of Germany, short-term stays for sporting events in endemic regions cannot be excluded. A vaccination history could be ruled out. The IgM-positive horse from Thuringia, in the central part of Germany (case no. 7), is consistent with the occurrence of WNV in this region. The first horse with a WNV infection was detected in 2019 [39], and in 2020, several WNV cases were found in resident and zoo birds [42]. Interestingly, this unvaccinated horse was hospitalized for the extraction of a cheek tooth due to alveolitis and showed no clinical signs of WNV. The serology results showed only a weak positive result in the IgM-ELISA but were negative in the IgG ELISA and VNT. This could be a rare event of a very recent WNV infection, where only an IgM titer was present at the moment of sampling [14]. Unfortunately, a follow-up serum sample was not available.

We could not find a statistically significant difference in the distribution of breed and sex between WNV positive and negative horses. Furthermore, even if ponies were slightly less likely to be seropositive in the univariate logistic model, the likelihood ratio test showed that this predictor was no longer important in the multivariate approach. Our results support a previous study [61] that did not find a sex or breed predisposition. In addition, they found a negative correlation between seropositivity and age. Their explanation for this correlation was the fact that younger horses might travel more often and to regions of high prevalence, increasing their risk of exposure [61]. Unfortunately, this could not be assessed in our study because no travel history data were available at the time of analysis. Additionally, the predisposition of age could not be confirmed in our study although the seropositive horses were younger because age was correlated to the site of sampling as horses in panel A (BE/BB) were younger than in panel B (NRW).

In about 50% of cases, recent exposure to WNV as indicated by IgM positivity, corresponded with clinical signs (Table 4), as described in both WNV lineage 1 and 2 infected horses [16,17,18,19,20,21,22]. However, frequently reported clinical findings in this study, including muscle fasciculation, hyperesthesia, or altered skin sensibility, are less frequently reported in association with a WNV infection (summarized by [14,16,17,18,19,20,21,22,23,62]). In our study, these signs occurred alone or in combination with the more commonly reported ataxia, gait deficit, or reduced proprioception in affected horses. In accordance with previous findings [14,16,17,18,19,20,21,22], fever was reported in only one of the cases. In one case (no. 5, Table 4) the neurological signs rapidly worsened, and the horse became recumbent and had to be euthanized. The presence of these neurological signs and the fatal outcome confirms the neuropathogenetic potential of WNV lineage 2 as described in Austria and Hungary [22,63].

In contrast to the predominance of typical neurological signs, one case (no. 1, Table 4) had been presented for recurrent abdominal pain and behavioral changes as the sole and unspecific neurological abnormality [60]. The presence of colic or obstipation of the large intestine has been reported with WNV lineage 2 in Austria [22] and might have to be added to the panel of possible clinical manifestations of a WNV infection [24]. One other case (no. 2, Table 4) was presented for cough, nasal discharge, and recurrent fever. In this case, the final diagnosis was respiratory tract infection for an unknown reason as no infectious agent could be isolated from the analyzed tracheal wash. A third horse (case no. 7) was hospitalized due to a dental problem, without any relevant WNV-associated clinical signs. In all these cases, the IgM positive results were detected retrospectively (case no 2, 4, 7, and also 8), and the reason for presentation and the final diagnoses were not related to WNV infection. These cases are probably similar to asymptomatic cases, as it is well known that only a small part of infected horses show WNV-specific clinical signs [13]. Furthermore, it is possible that neurological signs were too subtle or short-term to have been noticed by the owner.

Our IgG ELISA results were generally in concordance with the VNT results, demonstrating mostly anti-WNV-specific antibodies in ELISA-positives, but also few (*n* = 9) with cross-reacting antibodies related to TBEV or USUV. Only seven of the reactive sera (*n* = 86) could not be confirmed by VNTs, two sera from NRW and five from BE/BB (including three doubtful ELISA sera). Although VNT is the gold standard in identifying WNV-specific or cross-reacting antibodies, not all ELISA samples can be confirmed [48]. Generally, VNTs have a higher level of specificity but are less sensitive than ELISAs [64], as both methods measure different serological parameters, i.e., binding IgG antibodies in the ELISA as opposed to virus-neutralizing antibodies in the VNTs. Due to cross-reactivity, the circulation of known or as yet unknown flavivirus(es) in our regions cannot be excluded, but this is considered highly unlikely. Known flaviviruses that can induce cross-reactive antibodies, e.g., Louping ill virus [65], and Bagaza virus [66], as well as Meaban virus or Japan encephalitis virus, have not been found in Germany yet (summarized by [67]).

It has been shown before that horses are suitable as sentinels to detect the occurrence of WNV in an area (e.g., [21,61,68]). However, such sentinel studies can only provide meaningful results in areas without a TBEV history and WNV vaccinations, the latter, because antibodies after WNV infection cannot be distinguished from vaccination-derived antibodies. In order to directly reveal TBEV cross-reactions, it may be helpful to use a TBEV antibody ELISA [69] in parallel. The use of this assay in horses may even be useful to reveal TBEV endemic areas [48,70]. In our panel, the TBEV antibody-positive horses showed no typical neurological signs for TBEV as described by a few TBEV clinical cases [70,71]. Instead, the serum titers in our affected horses were detected retrospectively during a routine blood screening due to endocrinological or abdominal (colic) problems as well as pulmonary syndrome or lymphangitis. Seroconversion without any specific clinical signs of a TBEV infection has been described in certain livestock species, such as cattle, small ruminants, and pigs. A high seroprevalence rate (21.6%) for TBEV in horses has also been shown in Austria [72]. For Germany, it was demonstrated that grazing animals, particularly small ruminants and horses, are suitable sentinels for the detection of TBEV foci by serological surveillance [70,73]. Furthermore, the investigation of rodents is a very good tool for the spatial presentation of virus circulation (summarized by [74]). Furthermore, in some regions where horses are vaccinated against WNV, mules or donkeys could also be a useful tool in the epidemiological monitoring of WNV [75]. 

Reports about USUV infection in horses are very rare, and to date, only based on serological data (summarized by [76]). In Italy, the percentage of USUV seropositive horses was 89.2% in 2008 but only 7.8% in 2009 [77]. In Poland, a high seroprevalence of antibodies against USUV (27.98%) was found. In Croatia, USUV neutralizing antibodies were detected in two of 69 WNV ELISA-reactive horse serum samples [78], whereas, in Serbia, in 2009, one of 349 horses tested positive [79]. Our study is the first report of specific USUV neutralizing antibodies in two horses in Germany, although with low neutralizing titers (15 ND_50_). It is elusive why only a few seropositive horses with USUV-specific antibodies were detected since virus circulation appears to be increasing in Germany [54,58,80,81]. Recently co-infections with both viruses were detected in five dead birds [59] in the Berlin region where WNV and USUV are co-circulating. While these findings suggest that a high infection pressure of both viruses is present in this area, this cannot be corroborated by our results (panel A) to date. Further studies in horses are necessary to gain a better understanding of the circulation of USUV in the German horse population.

## 5. Conclusions

Our study demonstrated a high WNV seroprevalence rate in the horse population in Berlin/Brandenburg. Future serosurveys of horse populations in areas previously assumed to be unaffected will help to reveal the spread of the virus at an early stage. As long as the vaccination rates for WNV in these areas remain on a low level, horses can be important sentinels. When neurological signs occur in horses or nonspecific signs of unclear etiology, such as the presence of colic or obstipation of the large intestine in combination with high mosquito abundance, WNV infection should be included as an important differential diagnosis. When performing seroepidemiological studies, ELISA-positive sera should be further characterized by flavivirus-specific neutralization assays.

## Figures and Tables

**Table 1:** Reason for presentation and final diagnosis of the subcategory internal medicine-related disease in the sampled horses from the regions Berlin/Brandenburg (BE/BB/ panel A) and North Rhine–Westphalia (NRW/ panel B). Since the majority of typical WNV symptoms were found in the internal medicine-related disease (IM) category, only this one was further broken down into subcategories in the table.

Presented For	BE/BB 2019	BE/BB 2020	BE/BB Total	NWR 2018	NWR 2020	NWR Total	Total 2018–2020
Internal medicine-related disease (IM)	51	139	**190**	36	52	**88**	**278**
Musculoskeletal problem (MS)	39	46	**85**	10	14	**24**	**109**
Dental problem (Dental)	1	9	**10**	0	2	**2**	**12**
Health control (Healthy)	7	2	**9**	21	8	**29**	**38**
Total per year	**98**	**196**	**294**	**67**	**76**	**143**	**437**
**Final diagnosis of internal medicine-related disease**							
Suspected or diagnosed infectious disease	5	5	**10**	8	3	**11**	**21**
Clinically diagnosed WNV infection	1	3	**4**	0	0	**0**	**4**
Neurological disease	3	3	**6**	1	4	**5**	**11**
Gastrointestinal disease	23	85	**108**	6	9	**15**	**123**
Suspected or diagnosed intoxication	4	0	**4**	0	0	**0**	**4**
Respiratory tract disease	3	18	**21**	8	3	**11**	**32**
Cardiovascular disease	0	3	**3**	0	1	**1**	**4**
Anemia	1	0	**1**	0	0	**0**	**1**
Urogenital disease	6	8	**14**	1	0	**1**	**15**
Dermatological disease	0	3	**3**	3	6	**9**	**12**
Endocrinological disease	0	0	**0**	8	26	**34**	**34**
Suspected or diagnosed Neoplasia	1	1	**2**	0	0	**0**	**2**
Ophthalmological disease	4	10	**14**	1	0	**1**	**15**
Total per year	**51**	**139**	**190**	**36**	**52**	**88**	**278**

**Table 2:** Results of virological and ELISA testing of horse sera from panel A region Berlin/Brandenburg (BE/BB) and panel B North Rhine–Westphalia (NRW).

Sampling Year	Region	Panel	Total Number	RT-qPCR WNV (Pos./Total)	RT-qPCR USUV (Pos./Total)	RT-qPCR TBEV (Pos./Total)	IgG WNV ELISA (React./Total)	IgM WNV ELISA (Pos./Total)
2019	BE/BB	A	98	0/98	0/98	0/98	14/98	2/98
2020	BE/BB	A	196	0/196	0/196	0/196	32/196	6/196
2018	NRW	B	67 *	n.t.	n.t.	n.t.	2/67	0/67
2020	NRW	B	76	0/76	0/76	0/76	4/76	0/76

Abbreviations: n.t., not tested, react., reactive (positive and doubtful by IgG ELISA), pos., positive, * one donkey included.

**Table 3:** Results of the reactive ELISA horse sera (positive and doubtful) by different VNTs (range of the ND_50_ neutralization titers in brackets).

Year/Panel	Region	Reactive IgG WNV ELISA Samples Tested by VNTs	Confirmed by WNV-VNT (ND_50_)	Confirmed by TBEV-VNT (ND_50_)	Confirmed by USUV-VNT (ND_50_)	WNV Seroprevalence (%)	95% CI
2019/A	BE/BB	14	8 (10–1920)	1 (320)	1 (15)	8.16	3.59–15.45
2020/A	BE/BB	32	27 (10–960)	3 (10–480)	0	13.77	9.27–19.41
2018/B	NRW	2	0	2 (20,60)	0	0	0
2020/B	NRW	4	2 (80)	2 (15,80)	0	2.63	0.32–9.18

Abbreviations: BE/BB = Berlin/Brandenburg; NRW = North Rhine–Westphalia; CI = confidence interval.

**Table 4:** Summary of observed clinical signs during the hospitalization period of WNV IgM ELISA-positive horses from panel A (BE/BB), presentation of the relevant clinical signs similar to [22].

		2019		2020					
	Case No.	1	2	3	4	5	6	7	8
	ID	P 2028	P 2044	P 2167	P 2168	P 2197 *	P 2221	P 2244	P 2258
**Clinical signs**									
- **Neurological**									
Anxiety		A							
Increased excitability		A							
Bruxism						x	A		
Muscle fasciculation		x		x		x	x		
Hyperesthesia		x		x		x	x		
Increased sensibility to noises		x							
Unspecific lameness						A			A
Ataxia						x	x		
Reduced postural reaction				x			x		
Weakness				A					
Recumbency						x			
Cranial nerve deficits						x			
- **Unspecific**									
Fever (single)			x	A			A		
Fever (recurrent)			A						
- **Respiratory tract related**			A						
- **Gastrointestinal**									
Acute abdominal pain		A			x ^a^				
- **Musculoskeletal**									x ^b^
- **Dental**								x ^c^	

Abbreviations: x = signs during hospitalization period; A = signs reported only in the anamnesis; ^a^ lipoma pendulans; ^b^ fracture of the coffin bone; ^c^ Alveolitis; * had to be euthanized.

## Data Availability

The data that supports the findings of this study are available in the main manuscript and the Supplementary Materials of this article.

## Supplementary Materials

The following are available online at https://www.mdpi.com/article/10.3390/v14020243/s1, Supplementary Materials Data: Figure S1: Pie charts showing the distribution of breeds in the sampled horses, Figure S2: Pie charts showing the distribution of sex in the sampled horses, Table S1: detailed ELISA and different VNT results of each horse sample from panel A (BB/BE) from 2019, Table S2: detailed ELISA and different VNT results of each horse sample from panel A (BB/BE) from 2020, Table S3: detailed ELISA and different VNT results of each horse sample from panel B (NRW) from 2018, Table S4: Detailed ELISA and different VNT results of each horse sample from panel B (NRW) from 2020, Table S5: Detailed ELISA and different VNT results of vaccinated horses in panel A (BB/BE) in 2020, Table S6: Detailed ELISA and different VNT results of vaccinated horses in panel B (NRW) in 2020.
