# Supplementary material for: Seroepidemiological Survey of West Nile Virus Infections in Horses from Berlin/Brandenburg and North Rhine-Westphalia, Germany
Source: Viruses. 2022 Jan 25;14(2):243. doi: 10.3390/v14020243 (PMC8877243; doi:10.3390/v14020243)

Supplemental Data

Analyses of the specific breed and sex in the included population

The demographic distribution concerning breed and sex is shown in Supplemental Figures 1a and 2a. The detailed demographic distribution concerning breed per year and panel is pictured in Supplemental Figures 1b, 1c, 2b and 2c.

The Fisher's exact test did not reveal a statistically significant difference in breed distribution between the populations NRW 2018 and 2020 ($p = 0.50$), BE/BB 2019 and 2020 ($p = 0.38$) or between NRW and BE/BB in total ($p = 0.07$). See Figures 1a, b and c.

The Fisher's exact test did not reveal a statistically significant difference in sex distribution between the population NRW 2018 and 2020 ($p = 0.76$) or between BE/BB 2019 and 2020 ($p = 0.14$). However, the distribution of sex differed between NRW and BE/BB ($p = 0.001$). Indeed, in NRW almost no intact males were included. When comparing the distribution of males (including castrated and intact male) and females, the Fisher's exact test was non-significant for all comparisons (NRW 2018 to 2020 $p = 0.59$, BE/BB 2019 to BE/BB 2020 $p = 0.69$, NRW to BE/BB $p = 0.56$). See Figures 2a, b and c.

Figure S1:

S1a. Pie chart showing the distribution of **breeds** in the sampled horses **from panel A (BE/BB) and panel B (NRW)**. AQH-type, American quarter horse-type; DH, Draft Horse; STB, Standardbred; THB, Thoroughbred; WB, Warmblood.

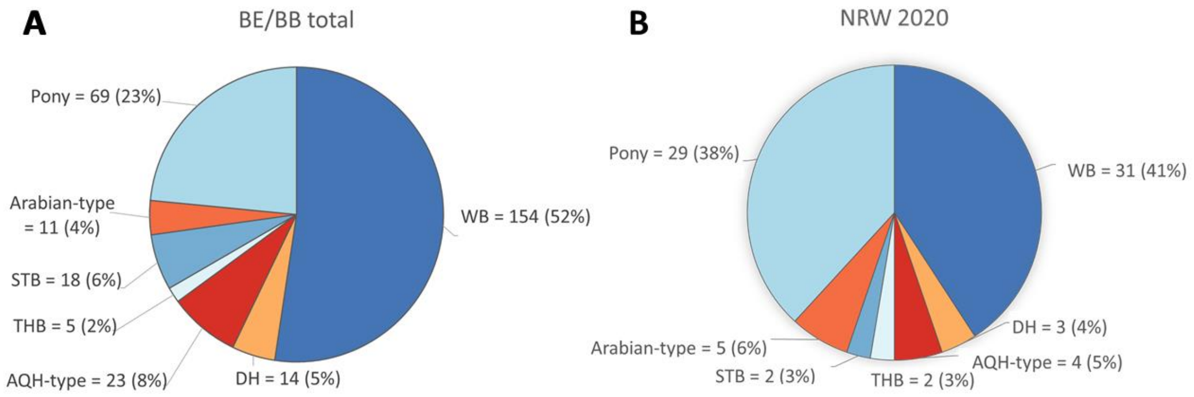

S1b. Pie chart showing the distribution of breeds in the sampled horses **from Berlin/Brandenburg (BE/BB) from 2019 (panel A1) and 2020 (panel A2).** AQH-type, American quarter horse-type; DH, Draft Horse; STB, Standardbred; THB, Thoroughbred; WB, Warmblood.

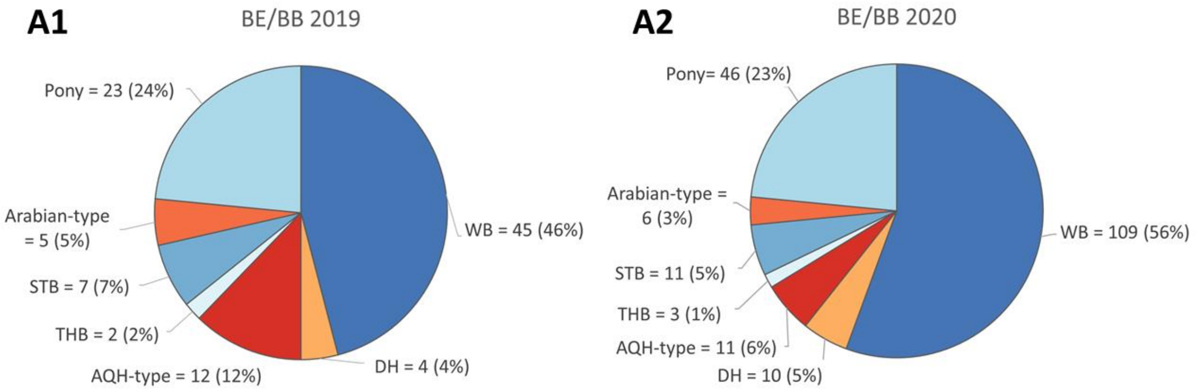

S1c. Pie chart showing the distribution of breeds in the sampled horses from **North Rhine-Westphalia (NRW) from 2018 (panel B1) and 2020 (panel B2)**. AQH-type, American quarter horse-type; DH, Draft Horse; STB, Standardbred; THB, Thoroughbred; WB, Warmblood.

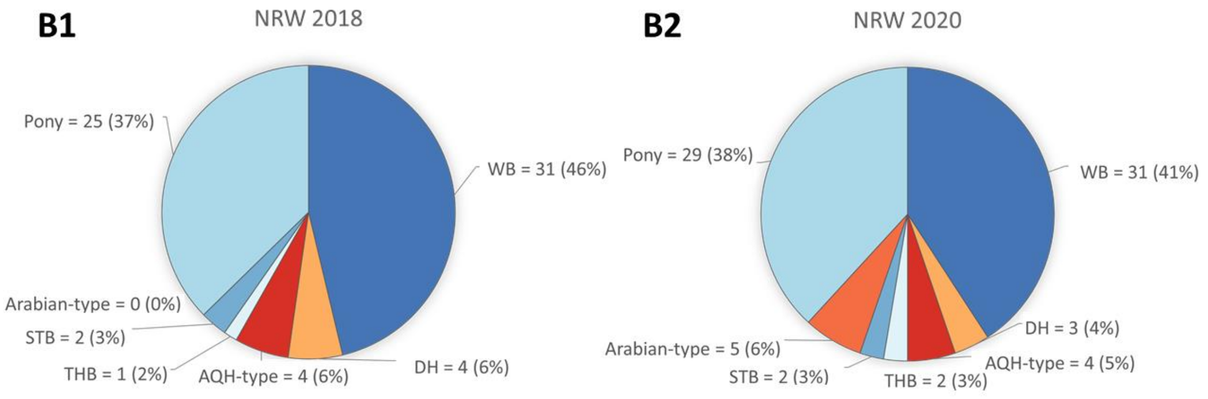

Figure S2:

S2a. Pie chart showing the distribution of **sex** in the sampled horses **from panel A (BE/BB) and panel B (NRW)**. F, female; C, castrated male; M, intact male; uk, unknown

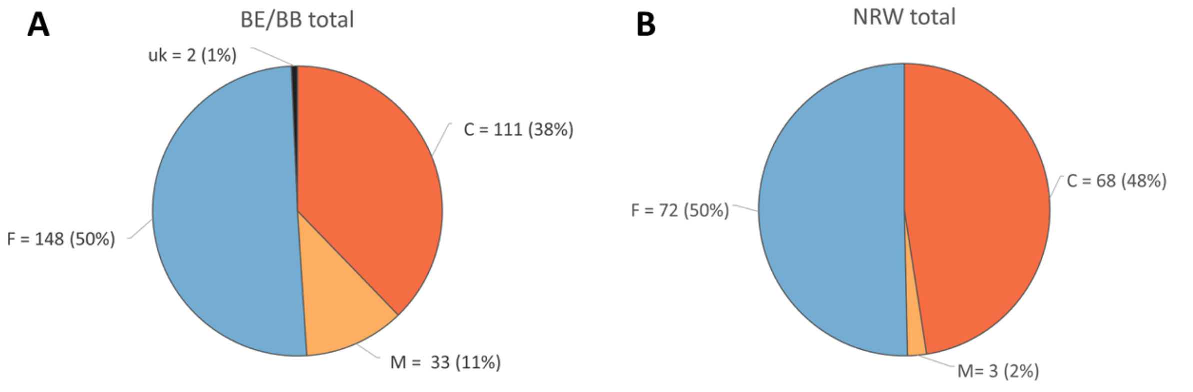

S2b. Pie chart showing the distribution of sex in the sampled horses from **Berlin/Brandenburg (BE/BB) from 2019 (panel A1) and 2020 (panel A2)**. F, female; C, castrated male; M, intact male; uk, unknown.

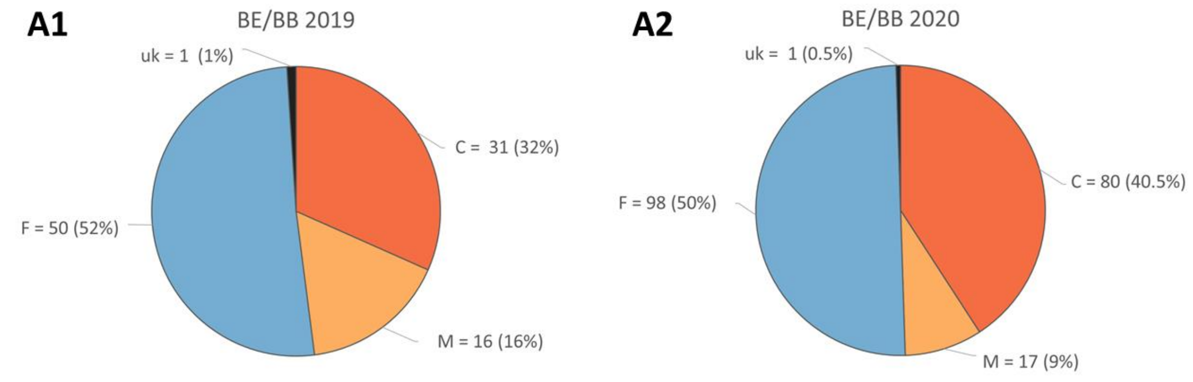

S2c. Pie chart showing the distribution of sex in the sampled horses from **North Rhine-Westphalia (NRW) from 2018 (panel B1) and 2020 (panel B2)**. F, female; C, castrated male; M, intact male; uk, unknown.

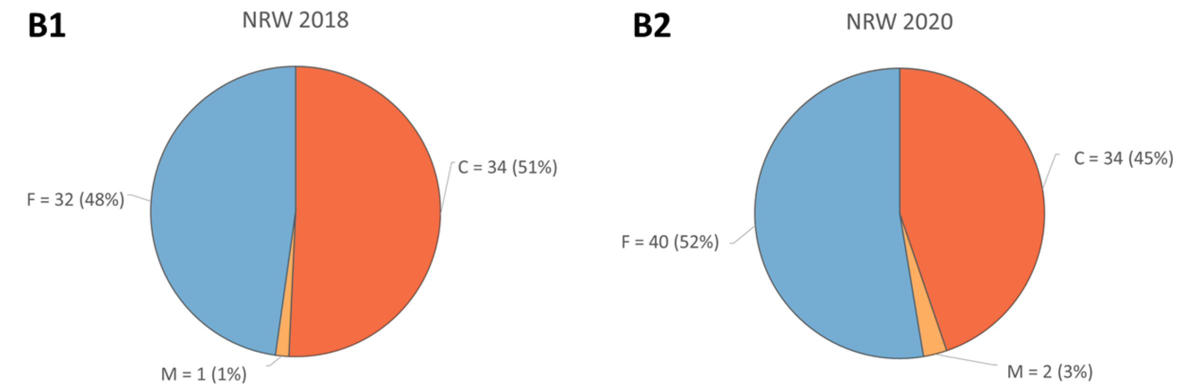

Supplement: Supplementary file 1 [file viruses-14-00243-s001.zip › Supplemental Data_Figures.pdf]
